# Supplementary material for: Cardiac, respiratory, and gastric rhythms independently modulate motor corticospinal excitability in humans
Source: PLoS Biol. 2025 Nov 12;23(11):e3003478. doi: 10.1371/journal.pbio.3003478 (PMC12629470; doi:10.1371/journal.pbio.3003478)
Supplement: Attachment — Submitted filename: Response_letter_PLOSBIO_FINAL.docx [file pbio.3003478.s006.docx]

We would like to thank all three reviewers on their thorough and helpful feedback on our manuscript. All changes based on the comments have been tracked in blue in the manuscript, and pasted in the responses where relevant.

*Reviewer #1 (Micah Allen): This is an insightful and well-executed study that addresses a complex and highly relevant question in neuroscience: how different internal bodily rhythms simultaneously and independently influence brain function, specifically motor corticospinal excitability. The manuscript is clearly written, and the methodologies employed are largely rigorous and appropriate for the research questions. The simultaneous investigation of cardiac, respiratory, and gastric influences on motor evoked potentials (MEPs) represents a novel and important step forward for the field of interoception and brain-body interactions. The findings have significant potential impact, particularly the demonstration of independent modulation by these three rhythms and the concept of individual "interoceptive profiles" that are not captured by self-report measures. The experimental design is careful (e.g., continuous neuronavigation, large trial counts), the statistics are modern and
transparent, and the paper stands to unify three organ systems within one protocol, demonstrating their functional independence, which will likely be of broad interest.

The study aimed to determine whether and how the cardiac, respiratory, and gastric visceral rhythms simultaneously and independently modulate corticospinal excitability in humans. The implicit hypothesis was that all three rhythms would show a coupling with MEP amplitude, and that these couplings would be independent of each other, suggesting distinct modulatory pathways. The authors successfully demonstrated that the phase of all three investigated visceral rhythms—cardiac, respiratory, and gastric—significantly modulates MEP amplitude, with similar effect sizes observed at the group level. A key finding was the independence of these modulatory effects: the strength of an individual's viscero-motor coupling for one rhythm did not correlate with their coupling strength for the other two. This independence was further substantiated by a GLM analysis which revealed no significant interaction terms between the influences of the different rhythms. These results strongly suggest
that distinct mechanisms underlie the coupling of each visceral rhythm with motor excitability, resulting in unique "interoceptive profiles" for each participant. Additionally, the study found that these objectively measured interoceptive coupling strengths were not correlated with participants' self-reported awareness of their internal organs, as assessed by the THISQ and MAIA-2 questionnaires. Finally, the study also confirmed that TMS coil-to-target distance significantly impacted MEPs, a factor that was appropriately accounted for in the analyses.

The study is of high scientific quality. Its novelty lies in the simultaneous assessment of these three key visceral rhythms and their independent effects on a well-established measure of corticospinal excitability. The use of neuronavigated TMS, comprehensive physiological recordings, and sophisticated statistical analyses (including circular spline GLMs and permutation testing) lends considerable strength to the findings. The paper makes a compelling case for refining current functional frameworks of interoception to account for such independent, multi-organ influences and individual variability.

Major Issues
While the study is robust and the conclusions are generally well-supported, a few areas warrant further attention to enhance its clarity and rigor:

1. The statistical significance of some primary outcomes requires careful consideration regarding multiple comparisons. Specifically, the reported p-values for the coupling strengths of the cardiac and gastric rhythms (p=0.04 for both, from one-sided Wilcoxon signed-rank tests) would not meet conventional significance thresholds if a standard Bonferroni correction for the three primary hypotheses (cardiac, respiratory, gastric) was applied. The manuscript should address this, either by incorporating such an adjustment and discussing its impact on the findings, or by providing a clear rationale for the current statistical approach, perhaps with increased emphasis on the interpretation of the provided Bayes Factor values.*

We agree that the full rationale for the statistical tests were not sufficiently clear in the original manuscript, and have therefore rewritten this section (2.8.3) of the methods to justify our approach.

Importantly, large r-values could be obtained by chance. To estimate chance level, we adopted the same approach previously used to demonstrate coupling between the phase of the gastric rhythm and the amplitude of neural alpha oscillations (57). In practice, for each participant, we created chance-level coupling by randomly shuffling the link between (cardiac, respiratory, or gastric) phases and MEP amplitudes, ran the model described above, and compute the r-value for this specific permutation of the data. We repeated this procedure to obtain 10.000 r-values obtained by chance. The participant’s coupling strength was then computed by subtracting the median of the permuted r value distribution from the empirical r value. We then tested whether at the group level, coupling strengths were significantly larger than zero using a one-sided Wilcoxon signed-rank test against zero. Here, we test whether empirical coupling was higher than permuted chance level, and thus one-sided tests were used. Of note, while coupling strength might occasionally be negative (but small) in a given participant, at the group level two signals cannot be significantly less phase-amplitude coupled than what would be expected by chance. Testing the other side of the hypothesis (empirical coupling lower than chance) would thus not be justified.

Because circular spline GLMs allow for only one circular regressor, we thus tested each visceral rhythm separately. Differences in SNR between the different visceral signals (i.e. sensitivity to accurately detect the phase of each rhythm at the time of the pulse) might impact the accuracy of the spline model. However, potential differences in the quality of phase estimation are also taken into account when estimating chance level per participant per visceral rhythm. The final index we used, coupling strength, is thus relatively immune to SNR and phase extraction issues. To correct for the effect of running one test per visceral rhythm, and this potentially inflating p-values, we applied an FDR correction to the obtained p-values.

Page 11, lines 334-357.

Furthermore we have now applied FDR corrections on the p-values of the circular models to correct for multiple tests being performed, and the statistics have been updated accordingly. FDR correction leaves main results and conclusions unchanged. Notably:

At the group level, we found coupling strengths to be significantly larger than zero for the cardiac (one-tailed Wilcoxon signed-rank test against zero, *z* = 1,79, *p_FDR_* = 0.047, BF_10_ = 3.84, Hedge’s g = 0.55), respiratory (*z* = 2.65, *p_FDR_* = 0.012, BF_10_ = 22.86, Hedge’s g = 0.54), and gastric (*z* = 1.67, *p_FDR_* = 0.047, BF_10_ = 2.87, Hedge’s g = 0.50) rhythms (see figure 2B).

Page 14, lines 460-464.

*2. The analysis of MEP amplitude involved first residualising it against coil-to-target distance, based on the assumption that variance related to coil distance is purely artifactual. However, as respiratory movements can cause some of the largest coil displacements, this regression could potentially remove a physiological component of interest if breathing-related posture changes also have a direct neural influence pathway. To confirm the robustness of the findings against this assumption, it would be beneficial to discuss this possibility or, ideally, provide a supplementary analysis (e.g., repeating the circular-spline GLM without this regressor, or including it as a linear covariate) to demonstrate that the three rhythm effects persist.*

We thank the reviewer for raising this important point. Our rationale for residualizing MEP amplitudes against coil-to-target distance was to reduce the risk of false positives. Respiratory movements can cause systematic coil displacements, which in turn alter MEP amplitude independently of neural excitability. Without controlling for this, apparent phase-locked changes in MEPs could simply reflect head-coil movement artefacts. Therefore, the aim of the performing all main analyses on MEP residuals was to increase statistical sensitivity, and reduce the chance of false positives. We agree with the reviewer that this conservative approach could also remove a genuine physiological component if breathing-related posture changes exert direct neural influences. We have clarified this point in the revised Methods. Importantly, to demonstrate robustness, we re-ran the circular spline GLM for respiration on the raw (non-residualised) MEP amplitudes and obtained similar results. Thus, while our chosen correction likely underestimates the physiological contribution of respiration, the main conclusions remain unchanged.We have clarified this now more clearly in the methods section. We further acknowledge that our approach, while being an excellent control against false positive, would also remove a physiological component related to breathing movement.

The distance of the TMS coil to the target stimulation site in the motor cortex is known to influence MEP amplitude and previous work has suggested controlling for such displacements increases statistical sensitivity to detect effects (54,55). This distance varies with experimenter and/or participant movement and might thus be in particular related to the participant’s breathing movement, which would confound all further analysis. Here, to reduce confounding effects of coil displacements and increase statistical power, we account for the effect of displacement of the TMS coil with respect to the participant’s head. By modelling the influence of distance-to-target on the MEP amplitude, we in particular correct for the effect of head displacement caused by the participant’s respiratory rhythm contributing to phase locked amplitude changes. Note that this conservative approach is also likely to remove any physiological component related to breathing-related movement.

Pages 9-10, lines 288-298.

Additionally, have added the results of running the model on the the raw z-scored MEP amplitudes to the results section:

To ensure the circular model on respiratory phase was not biased by the distance-to-target correction (i.e. running the model on the MEP residuals), we re-ran this model using the z-scored raw MEP values, and obtained similar results (*z* = 1.92, *p*= 0.027, BF_10_ = 3.02, Hedge’s g = 0.49).

Page 14-15, lines 468-471.

*3. The conclusions regarding the independence of coupling strengths among the three rhythms, and the lack of correlation with questionnaire data, are primarily based on non-significant findings from correlation analyses. Given the sample size of N=28, the statistical power to detect small to moderate correlations is limited. A more explicit discussion of this limitation is warranted, for instance by noting that "evidence for absence was modest" where appropriate, particularly when interpreting these null results and considering the Bayes Factors, some of which were inconclusive (e.g., for cardiac and respiratory THISQ correlations). It might also be mentioned in the discussion that future studies should supplement this by utilizing psychophysical measures of objective interoception.*

We agree that these are limitations of this study which were insufficiently acknowledged in the original manuscript. These limitations have now been explicitly added in the discussion:

Despite this, we still found an absence of correlation between self-reported interoception and objectively measured interoceptive influence on motor excitability. It has to be noted that the sample of the current study might not be sufficiently large for the detection of small correlational effects. Whereas the Bayes Factor indicates some support for the null for the correlation between the THISQ gastric and gastric coupling strength, the Bayes evidence for the other two viscera was inconclusive. Whether and how objective and subjective measures of interoception relate to each other remains an open issue, with potential clinical relevance (3). Here, future studies would benefit from larger sample sizes and the inclusion of subjective, psychophysical, and neural measures of interoception simultaneously, to further probe the independence of these various levels of interoception. In parallel, more work is needed to fine-tune analysis approaches incorporating phase effects of multiple visceral rhythms at once, and their sensitivity to pick up on the different ways in which these effects could manifest (e.g. binary versus circular effects, as well as different coupling modes (98)).

Page 22, lines 707-720.

*4. The methodology for testing interactions using GLM Model 2 involved binarizing visceral phases into "excitatory" and "inhibitory" periods based on each individual's MEP data. While this approach allows for the inclusion of all three rhythms in a single model, its data-driven nature, which enforces main effects as acknowledged by the authors, and its simplification of continuous phase information, should be more thoroughly discussed. An expanded rationale for choosing this method, along with a consideration of how it might affect sensitivity to detect certain types of interactions, would be beneficial. Furthermore, a brief discussion point acknowledging that future work might explore alternative approaches, such as GLMs incorporating sine and cosine transformed continuous phase angles to model interactions without binarization, could add valuable perspective on methodological advancements.*

The choice for a binarized GLM was to support the correlation analysis between coupling strengths, rather than a main analysis in and of itself to support the conclusion of independence. We agree that this was not entirely clear in the original manuscript, and have now clarified this point throughout the manuscript.

In the results:

At the participant’s level, do we find evidence for interoceptive-sensitive or interoceptive-insensitive participants? In other words, is high coupling with one visceral rhythm predictive of high coupling with another visceral rhythm? We used two different approaches to test for potential independence. Firstly, we tested for correlations between the individual coupling strengths of the three visceral rhythms with each other (Figure 2C). We found no correlation between coupling strengths, with all tested correlations providing moderate evidence for the null hypothesis (Kendall’s tau B correlations, all *p* values > 0.49, all BF_10_ < 0.33). By exploring the interoceptive profiles of the participants with the highest coupling strength of each rhythm, it is apparent that high coupling with one rhythm does not necessarily mean high or even any coupling with another rhythm, and all types of interoceptive coupling profiles can be possible, as illustrated in figure 2D and see supplementary figure 1.

We confirmed those results using a second approach, where all rhythms were combined in the same general linear model. To switch from a circular spline model to a general linear model with interactions between rhythms, we defined for each participant and each rhythm an interval of pi where MEP amplitude was maximal (excitatory phase), and an interval of pi where MEP amplitude was minimal (inhibitory phase). For each participant, we then accounted for MEP amplitude by binarized phases for all three rhythms, and, importantly, all possible interactions between rhythms. The model confirms a significant influence of all three rhythms, as expected by the model design, but revealed an absence of interactions between rhythms: We found no double or triple interactions between any of the visceral phases (all *p* values for interaction terms > 0.18). Except for the interaction term between cardiac and gastric phases (BF_10_ = 0.47), all BF_10_ values indicate moderate evidence for the null hypothesis for all other interactions (BF_10_ < 0.33).

Together, these two separate analyses converge on the same conclusion, which is that even though the phase of each of the three visceral rhythms influences the amplitude of the MEP, they do so in an independent manner: at the participant’s level, high coupling between motor excitability and one rhythm provides no information of the coupling with another rhythm, resulting in a large variety of individual interoceptive coupling profiles

Page 15, lines 475-504.

And in the discussion:

Our results show that motor excitability, measured from the hand region in primary motor cortex down to a specific muscle, is independently related to the cardiac, respiratory and gastric rhythms. This independence was confirmed in two separate analyses: a correlation between individual coupling strengths between the different organs, and a binarized GLM in which interaction effects between the visceral rhythms were likewise absent. Besides the interaction terms between cardiac and gastric phase in the GLM, Bayes factors of all other tests provided moderate evidence for absence of an effect.

Page 18, lines 581-587.

We also agree with the reviewer that more work is still need on the different statistical approaches needed to carefully map these types of interactions, and have now acknowledged that in the discussion.

In parallel, more work is needed to fine-tune analysis approaches incorporating phase effects of multiple visceral rhythms at once, and their sensitivity to pick up on the different ways in which these effects could manifest (e.g. binary versus circular effects, as well as different coupling modes (98)).

Page 22, line 716-720.

*Minor Issues

- There appears to be a discrepancy in the PDF: the image on page 39 (labelled "Figure 3" in its file) seems to correspond to the text description for Figure 4 (which discusses phase-binned MEPs for cardiac and respiratory rhythms). Conversely, the image on page 40 (labelled "Figure 4" in its file) appears to correspond to the text description for Figure 3 (correlation with THISQ). Please verify and correct.*

Figure numbers have been corrected

*- The regression of coil-to-target distance is a crucial step. Consider reporting the mean (±SD) coil-to-target distance and perhaps its dominant frequency component or variability to show that residual respiratory or other movement-related artefacts were likely well-controlled by the regression.*

Information on mean(SD) coil distance of included trials has been added to the methods section:

Mean(SD) distance-to-target of included trials was 3.07(1.7)mm.

Page 6, line 160.

Calculating a dominant frequency component on the coil-to-target distance measure is unfortunately not possible, as it is a discrete measure only sampled at the time of the pulse.

*- Clarify if the individual stimulation intensity (set to elicit "approximately 1mV") was set relative to a percentage of resting motor threshold (RMT), or if RMT was not formally determined. The group mean ± SD %MSO is reported.*

This has been clarified in the methods:

Individual stimulation intensity was set to elicit an MEP of approximately 1mV in amplitude (mean stimulation intensity(SD) = 41(7)% maximum stimulator output). Motor threshold was not used to determine stimulation intensity.

Page 5, lines 146-149.

*- The manuscript reports the mean (SD) of trials remaining. Could a brief breakdown of the proportion of trials excluded for different reasons (low EMG amplitude, saturation, signal-to-noise ratio, coil displacement) be provided, perhaps in supplementary materials?*

We have added the information on how many trials were on average excluded due to noise in each of the recorded signals to the methods section:

Per participant, a mean(SD) number of trials were excluded due to either MEP amplitude or pre-MEP noise in the EMG (24.6(27.5)), distance-to-target (1.3(1)), cardiac signal (2.5(4.6)), respiratory signal (36.6(37.3=), or gastric signal (14.3(9.8)) (note that a trial was excluded if it failed inclusion criteria for one of the signals, but could be labelled for exclusion due to multiple signals showing issues at once).

Page 9, lines 268-272.

*Given that two participants required a relaxed artifact criterion for EGG and the generally lower SNR for EGG, please consider adding a brief note quantifying average cycle variability or another quality metric for the EGG data to allow readers to gauge the reliability of the gastric phase estimates.*

We have added information about EGG cycle variability in the EGG preprocessing section:

After preprocessing, the mean(SD) length of clean gastric cycles was 20.5(1.3) seconds, and mean standard deviation (SD) of participant’s gastric cycle was 1.95 (min SD: 0.99; max SD: 3.34).

Page 9, lines 263-265.

*- The authors rightly note the gastric rhythm has "no true zero point in the cycle". A brief sentence explaining how the circular spline GLM accommodates this (e.g., by focusing on the modulation shape irrespective of absolute phase origin) could be helpful for some readers.*

We have clarified this point in the methods section:

Given that the largest deviation can occur at any phase within a visceral cycle, and r values are computed per participant, this model thus does not require phase-alignment of coupling effects on the group level. Rather, we here tested whether in general MEP amplitudes are coupled to visceral phase higher than chance level (see below), irrespective of the phase in which each participant showed this coupling.

Page 10, lines 326-330.

*- Two left-handed participants were included in the final sample of 28. Please briefly justify their inclusion (e.g., stating that their data did not appear as outliers or that repeating analyses without them did not change the main conclusions) or acknowledge this as a minor source of heterogeneity.*

We verified that the coupling scores of the two left-handed participants were not outliers, and therefore biasing the results. This information has been added to the methods section:

It was verified that the two left-handed participants were not outliers, i.e. their coupling scores with each of the viscera did not deviate more than three standard deviations from the group mean.

Page 5, lines 125-127. *- 1000 permutations were used for the null distributions in the circular spline GLM. While the median of the permuted distribution (used for CS calculation) is likely stable, a brief note acknowledging that higher counts (e.g., 10,000) are sometimes advocated for very precise p-value estimation from permutations could be considered if recalculation is not feasible.*

We increased the number of permutations to 10.000 as suggested, and report the new values in the revised text and figures. Increasing the number of permutations did not have any noticeable effect on coupling strength estimation in any of the partipants.

*- The authors state "custom code and preprocessed data used for the analyses reported in this article are available through* [*osf.io/6utgf/*](http://osf.io/6utgf/)*". This is comendable, but do consider if anonymised raw physiological traces could also be uploaded to enhance reproducibility further, if ethically permissible.*

It is not ethically permissible to directly share human raw data on an open repository, however raw data can be obtained through a data sharing agreement with Maastricht University. We have clarified this point in the Data & Code availability section.

To comply with EU laws, individual data can only be shared once an institutional data sharing agreement with Maastricht University has been signed.

Page 24, lines 754-755.

*- Note in the Methods or Discussion whether female hormonal phase was recorded; if not, briefly acknowledge this as a potential unmeasured variable that can influence corticospinal excitability.*

We have added the following sentences to the discussion to acknowledge potential effects of hormonal phase on the here reported effects:

Moreover, current frameworks do not yet account for physiological changes occurring at even slower timescales, such as hormonal fluctuations during the menstrual cycle. Although not measured in the current experiment, previous work suggests effects menstrual phase on cortical excitability (90) as well as potential influences on heart-brain interactions (91).

Page 21, lines 672-676.

*- Typographical Errors:
"matrix sixe" should be "matrix size".*

Fixed

*"[z(Coil distance)]" has an unmatched closing bracket in GLM Model 1.*
Fixed
*- The discussion generally uses cautious language (e.g., "independently related," "contributed to motor excitability"). Ensure consistency in avoiding overly strong causal claims (e.g., "modulate" implies influence but ensure it's not taken as definitively proven direct causality from this observational design) where* *correlational or phase-amplitude coupling relationships are described.*

We have toned down the language of the abstract and discussion, as well as giving more fair acknowledgements of the limitations of the current study. For instance in the Abstract we changed “All three visceral rhythms contributed to motor excitability” into “All three visceral rhythms were coupled to motor excitability”.

*- It would be great to see a discussion of these findings in light of recent research on unimodal versus polymodal vagal sensory pathways (e.g., Chang et al., Nature, 2022,*[*https://www.nature.com/articles/s41586-022-04515-5*](https://www.nature.com/articles/s41586-022-04515-5)*). In particular, considering the differences in methodological scope could be insightful. While invasive animal studies offer greater spatial specificity, the current study provides valuable positive evidence for independent influences in humans. However, the discussion should likely maintain appropriate caution, acknowledging that this single study, focused on corticospinal excitability, may not exhaustively rule out all potential poly-organ or multi-organ interactions that might exist in other physiological or cognitive domains, or be detectable with different methodologies.*

Thanks for pointing out the very relevant work in mice, we have added to the discussion – not expanding further though, since our study is quite far from the mouse experiments (different species but also subcortical vs. cortical).

The independence of the effects suggests that each coupling could be mediated by at least partially distinct mechanisms. Our results do not allow to be more specific about the nature of the mechanisms, but it is worth reminding that there are quite a number of possibilities. Firstly, while all three organs are connected to the central nervous system through cranial nerves (vagal and glossopharyngeal) and spino-thalamic pathways (cardiac, phrenic, and splanchnic nerves), some of those pathways might be organ-specific, as recently shown in mice (59,60). Secondly, each rhythm also has specificities. For instance, cardiac contractions translate into blood pulsations that might be directly transduced in the brain by both neurons and glial cells (61,62), and respiration is detected by olfactory receptors acting also as mechanoreceptors, inducing neural oscillations in large cortical territories (63–66).

Page 19, lines 603-612.

We have also added a paragraph in the Discussion (also in response to comments by Reviewer 3) that notably highlighting the many unknowns in this field:

Evidence for organ-specific influences on brain signals in humans is still limited. Here, the focus was on excitability changes in the corticospinal motor system, and whether these findings extend to different physiological and cognitive states remains to be tested. Another remaining question is how viscera-viscera interactions moderate effects of visceral rhythms on the brain. Beyond the well-known heart rate modulation by the respiratory rhythm (respiratory sinus arrhythmia), most of the effects of viscera-viscera interactions are still unknown, and might appear as phase-rate coupling, phase-phase and phase-amplitude coupling. The exact nature of these complex interactions, and how they might impact the influence of visceral rhythms on MEPs needs to be addressed in future work.

Page 20, lines 639-647. *Addressing these points will further enhance the clarity, rigor, and impact of this already valuable contribution to the literature on interoception and brain-body dynamics.*

*Reviewer #2: Overview: 
This study aimed to examine how the excitability of the human motor system varies in relation to the phase of three visceral rhythms- cardiac, respiratory, and gastric. To this end, the authors applied single-pulse Transcranial Magnetic Stimulation (TMS) over the hand area of the primary motor cortex to elicit Motor Evoked Potentials (MEPs), while simultaneously recording visceral signals. They then assessed whether MEP amplitude depended on the phase of each visceral rhythm. The authors found that all three rhythms significantly modulated MEP amplitude, indicating a phase-dependent coupling between visceral activity and motor excitability. They also observed that the coupling strengths for each organ were independent of one another - individuals showing strong coupling with one organ did not necessarily show strong coupling with the others. Moreover, these objective coupling measures did not correlate with self-reported interoceptive awareness. The authors concluded that
these findings support the existence of distinct, organ-specific mechanisms underlying viscera-motor coupling and highlight the importance of considering individual interoceptive profiles when interpreting brain-body interactions.

Positive features of the study 
* Comprehensive, multi-level, and cross-organ investigation: The study examines not only the relationship between MEP amplitude and the phase of organ activity at the group level but also explores inter-individual variability. This approach highlights discrepancies between group-level and individual-level findings and underscores the importance of considering multiple levels of analysis in body-brain interaction research.
* Sophisticated analytical approach: The study employs advanced analytical methods, demonstrating a high degree of methodological rigor and sophistication in addressing the research question.
* Clear writing and engaging figures: The manuscript is well written, with a clear structure and accessible language that effectively conveys complex findings. The figures are visually engaging and thoughtfully designed, further enhancing the clarity of the results.*

*Major considerations for improvement of the manuscript include the following:
* Need for additional detail in methodological description: The study employs sophisticated data processing and modeling techniques; however, several methodological aspects lack sufficient detail for full comprehension and replication. Providing further information - such as visual examples of raw experimental data, detailed steps for identifying cardiac features (e.g., R-peaks, T-wave offset), and references supporting the computation of coupling strength - would enhance the clarity, accessibility, and reproducibility of the approach.*

We have updated figure 1 in accordance with these comments and also comments by reviewer 3. Figure 1 now depicts a 20 second segment of time taken from all recorded signals from the same participant, representing multiple cycles of the cardiac and respiratory rhythm, one cycle of the gastric rhythm and three evoked responses in the EMG, as well as adding a time scale for clarity. We have added in figure 3A a representation of the R-peak and end of T to add clarity on how the two phases of the cardiac cycle were calculated.

Additionally we have rewritten the section in which the identification of cardiac events is described to improve the clarity of the methods:

Continuous ECG data was band-pass filtered between 1 and 40 Hz (windowed-sinc FIR filter, one pass-zero phase shift). To identify the PQRST waves on the ECG, we followed the procedure described in (48). Briefly, for each participant and block, we establish a template of the cardiac cycle (by averaging a few prototypic cycles) and correlate the template with the continuous ECG time series. R-peaks were identified on the normalized correlation as exceeding a threshold of 0.6. Misidentified R-peaks were manually corrected. Further cardiac events (P, Q, S, and T waves) were defined relative to the identified R-peak as local minima or maxima in preset time windows. In particular, the peak of the T-wave was identified as the local maximum occurring within 420ms after the Q-wave.

We define systole as the time between R-peak and end of T-wave, and diastole as end of T-wave until the next R-peak (see figure 3A).

Noisy cycles (i.e. with an unclear T-wave) were identified by excluding any cycles in which the RT interval exceeded three standard deviations from the mean of that block. For the main analysis, instantaneous cardiac phase was calculated by assigning radian values between 0 and 2π to each TMS pulse based on its relative timing to the R-peak preceding the pulse and the first R-peak following the pulse.

To identify the end of the T-wave, we used the second derivative of the signal: a participant specific block-by-block mean T-wave was computed, and the local maximum in the second derivative of the mean T-wave between the peak of T up to +150ms was taken as end of the T-wave. Given the known low variability of the QT interval under resting conditions (standard deviation <5ms, (49)), a fixed duration of cardiac systole was calculated for each participant by taking the mean end of T time of the three blocks (i.e. mean R to end of T duration). Cardiac diastole was then defined as the remained of the cardiac cycle (i.e. end of T to the next R peak).

Pages 7-8, lines 213-239.

** Interpreting the functional significance of coupling strength: Although the coupling strengths were statistically greater than zero, their functional relevance remains unclear. While the use of one-sided tests may have contributed to statistical significance, it is still possible that the observed coupling does not reflect a meaningful functional interaction. Contextualizing coupling strength values relative to known benchmarks, if available, would help clarify their significance.*

We have significantly revised the methods section to clarify the use of one-sided tests, adding references on previous uses and interpretations of coupling strengths, and also based on suggestions from reviewer 1 have recomputed our circular spline models for more rigorous statistical interpretations (FDR correcting our p-values and rerunning our models with a higher number of permutations).

Importantly, large r-values could be obtained by chance. To estimate chance level, we adopted the same approach previously used to demonstrate coupling between the phase of the gastric rhythm and the amplitude of neural alpha oscillations (57). In practice, for each participant, we created chance-level coupling by randomly shuffling the link between (cardiac, respiratory, or gastric) phases and MEP amplitudes, ran the model described above, and compute the r-value for this specific permutation of the data. We repeated this procedure to obtain 10.000 r-values obtained by chance. The participant’s coupling strength was then computed by subtracting the median of the permuted r value distribution from the empirical r value. We then tested whether at the group level, coupling strengths were significantly larger than zero using a one-sided Wilcoxon signed-rank test against zero. Here, we test whether empirical coupling was higher than permuted chance level, and thus one-sided tests were used. Of note, while coupling strength might occasionally be negative (but small) in a given participant, at the group level two signals cannot be significantly less phase-amplitude coupled than what would be expected by chance. Testing the other side of the hypothesis (empirical coupling lower than chance) would thus not be justified.

Because circular spline GLMs allow for only one circular regressor, we thus tested each visceral rhythm separately. Differences in SNR between the different visceral signals (i.e. sensitivity to accurately detect the phase of each rhythm at the time of the pulse) might impact the accuracy of the spline model. However, potential differences in the quality of phase estimation are also taken into account when estimating chance level per participant per visceral rhythm. The final index we used, coupling strength, is thus relatively immune to SNR and phase extraction issues. To correct for the effect of running one test per visceral rhythm, and this potentially inflating p-values, we applied an FDR correction to the obtained p-values.

Page 11, lines 334-357.

** Caution in interpreting statistical independence: The conclusion that brain-viscera coupling is independent across the three visceral rhythms is based primarily on non-significant findings from generalized linear model (GLM) analyses. However, the absence of statistically significant interaction terms does not conclusively demonstrate functional independence. In my view, the current interpretation may overstate statistical non-significance as evidence of functional independence.*

The conclusions of independence of brain-viscera coupling effects was mostly supported by the correlation analysis, which provided moderate Bayes evidence in support of absence of evidence. The absence of interaction effects in the GLM was used as further support, taken together with the correlation analysis. We fully agree that this point was not sufficiently clear in the original version of the manuscript, and at several points throughout the manuscript have now clarified this. In particular, and also in response to comments by Reviewer #1, we have modified the presentation of the Results:

In the results:

At the participant’s level, do we find evidence for interoceptive-sensitive or interoceptive-insensitive participants? In other words, is high coupling with one visceral rhythm predictive of high coupling with another visceral rhythm? We used two different approaches to test for potential independence. Firstly, we tested for correlations between the individual coupling strengths of the three visceral rhythms with each other (Figure 2C). We found no correlation between coupling strengths, with all tested correlations providing moderate evidence for the null hypothesis (Kendall’s tau B correlations, all *p* values > 0.49, all BF_10_ < 0.33). By exploring the interoceptive profiles of the participants with the highest coupling strength of each rhythm, it is apparent that high coupling with one rhythm does not necessarily mean high or even any coupling with another rhythm, and all types of interoceptive coupling profiles can be possible, as illustrated in figure 2D and see supplementary figure 1.

We confirmed those results using a second approach, where all rhythms were combined in the same general linear model. To switch from a circular spline model to a general linear model with interactions between rhythms, we defined for each participant and each rhythm an interval of pi where MEP amplitude was maximal (excitatory phase), and an interval of pi where MEP amplitude was minimal (inhibitory phase). For each participant, we then accounted for MEP amplitude by binarized phases for all three rhythms, and, importantly, all possible interactions between rhythms. The model confirms a significant influence of all three rhythms, as expected by the model design, but revealed an absence of interactions between rhythms: We found no double or triple interactions between any of the visceral phases (all *p* values for interaction terms > 0.18). Except for the interaction term between cardiac and gastric phases (BF_10_ = 0.47), all BF_10_ values indicate moderate evidence for the null hypothesis for all other interactions (BF_10_ < 0.33).

Together, these two separate analyses converge on the same conclusion, which is that even though the phase of each of the three visceral rhythms influences the amplitude of the MEP, they do so in an independent manner: at the participant’s level, high coupling between motor excitability and one rhythm provides no information of the coupling with another rhythm, resulting in a large variety of individual interoceptive coupling profiles.

Page 15, lines 475-504.

And in the discussion:

Our results show that motor excitability, measured from the hand region in primary motor cortex down to a specific muscle, is independently related to the cardiac, respiratory and gastric rhythms. This independence was confirmed in two separate analyses: one showing an absence of correlation between individual coupling strengths between the different organs, and a binarized GLM in which interaction effects between visceral rhythms were likewise absent, with moderate Bayes evidence for absence of interactions in two interactions out of three.

Page 18, lines 588-594.

*Additional comments:

* Page 3 | Lines 79-81: The statement "Still, no experiment to date measured all three visceral rhythms: the vast majority of experiments are limited to one visceral rhythm, with few notable exceptions combining two (14-16)." may underestimate the number of studies examining cardiac, respiratory, and gastric signals together, especially outside the TMS field. If this limitation is specific to TMS-based studies, it would be helpful to make the scope of the claim more precise.*

We have reformulated this sentence to clarify that in general, it is very uncommon to measure multiple visceral rhythms at once, and that this limits our understanding of potential independent influences of visceral rhythms on brain dynamics. In other words, this is not specific to TMS studies.

Indeed, it is possible that visceral rhythms exert an influence in a co-dependent manner, for example through global changes in arousal states mediated through the vagus nerve (14). Still, this underlying assumption remains to be empirically tested. In the field of interoception, even if multiple organs are considered they are typically limited to cardiac and respiratory signals (15–19). When it comes to transcranial magnetic stimulation (TMS), only the cardiac rhythm has been measured so far (20–24). To date, no experiment measured all three visceral rhythms, leaving their potential independent influences on the brain speculative.

Pages 3-4, lines 79-86.

** Page 7 | Lines 191-192: The description of MEP amplitude as "the difference between the local maximum and local minimum between 20-60 ms after the pulse" would benefit from a reference supporting this methodological choice.*

We have added references and clarification to support this analysis choice:

MEP amplitude was calculated as the difference between the local maximum and local minimum (peak-to-peak) between 20-60ms after the pulse, falling well within the range in which MEP peaks typically occur (44,45).

Page 7, lines 200-202.

** Page 7 | Line 197: The acronym "RMS"  should be defined upon first use.*

Done

** Page 7 | Line 201: The method for R-peak detection, using a normalized convolution threshold of 0.6, would benefit from a supporting reference.*

We have rewritten the section on ECG preprocessing and added supporting references where needed.

** Page 8 | Line 244 - Reference to Liang et al. (2008): This paper primarily addresses variable/model selection, which may not align well with the statistical focus of the current study. A more suitable reference for Bayesian statistics could be Wagenmakers et al. Psychon Bull Rev 2018a, 2018b.*

Done

** Page 9 | Line 258: The term "z-scored peak-to-peak amplitude of the MEP" appears to describe the same measure as "difference between the local maximum and local minimum between 20-60 ms after the pulse" in Section 2.7. Consistent terminology across sections would improve clarity.*

We have clarified in section 2.7 that this indeed corresponds to the same data, and made the terminology aligned with the following sections.

MEP amplitude was calculated as the difference between the local maximum and local minimum (peak-to-peak) between 20-60ms after the pulse, falling well within the range in which MEP peaks typically occur (44,45).

Page 7, lines 200-202.

For all reported analyses, selected peak-to-peak MEP amplitudes were z-scored (see section 2.8.2).

Page 7, lines 210-211.

** Page 9 | Lines 281-282: The sentence "In the null model each data point corresponds to the mean of the data, generating data with a uniform distribution along phase" is unclear. Does "data" refer to MEP amplitude? If so, please clarify.*

This sentence has been rewritten for clarity:

The null model consists of data points whose values are set to equal the mean of the participant’s data, along the phase of the visceral rhythm, thereby creating a uniform distribution (where amplitude does not change as a function of phase).

Page 10, lines 321-324.

** Page 9 | Lines 283-284: The definition of the r-value suggests non-negative values. However, Figure 2B displays negative coupling strength values. Please clarify the computation and interpretation of the r-value.*

The interpretation of coupling strength values has been further clarified in the methods section, as detailed above in response to the Reviewer’s point on the functional interpretation of coupling strength. In particular,

To quantify phase-amplitude coupling the maximum absolute deviation between the spline and null model is calculated (r value). The larger the r value, the more the empirical data deviate from a uniform distribution. Given that the largest deviation can occur at any phase within a visceral cycle, and r values are computed per participant, this model thus does not require phase-alignment of coupling effects on the group level. Rather, we here tested whether in general MEP amplitudes are coupled to visceral phase higher than chance level (see below), irrespective of the phase in which each participant showed this coupling. We ran one model *per visceral signal* (thus three models) and *per participant* to obtain individual empirical r values.

Pages 10-11, lines 324-332.

Additionally, when presenting the Results, we clarify again this important point:

Using a circular spline GLM approach (56), we estimated, for each participant, how much MEP amplitude varies with the phase of each visceral rhythm. We computed coupling strengths, reflecting the difference between empirically obtained coupling scores and the median of chance level coupling scores obtained by permutation. At the participant level, a large positive coupling strength value indicates more coupling than expected by chance, while a small positive or negative coupling strength indicates chance level coupling. At the group level, we found coupling strengths to be significantly larger than zero for the cardiac (one-tailed Wilcoxon signed-rank test against zero, *z* = 1,79, *p_FDR_* = 0.047, BF_10_ = 3.84, Hedge’s g = 0.55), respiratory (*z* = 2.65, *p_FDR_* = 0.012, BF_10_ = 22.86, Hedge’s g = 0.54), and gastric (*z* = 1.67, *p_FDR_* = 0.047, BF_10_ = 2.87, Hedge’s g = 0.50) rhythms (see figure 2B). This result demonstrates that fluctuations of corticospinal excitability over time are driven by the phase of each of the three visceral rhythms, with similar effect sizes at the group level.

Page 14, lines 455-466

** Page 9 | Line 295: If coupling strength values may have negative values, it may be more appropriate to use a two-sided Wilcoxon signed-rank test rather than a one-sided test against zero.*

We have clarified the motivation for a one-sided test in the methods section. A negative coupling strength indicates that the participant’s empirical r value was lower than their permuted median value, however negative coupling strengths have no inherent functional interpretation (i.e. testing for lower coupling than chance level). Therefore, all reported tests are one-sided.

Importantly, large r-values could be obtained by chance. To estimate chance level, we adopted the same approach previously used to demonstrate coupling between the phase of the gastric rhythm and the amplitude of neural alpha oscillations (57). In practice, for each participant, we created chance-level coupling by randomly shuffling the link between (cardiac, respiratory, or gastric) phases and MEP amplitudes, ran the model described above, and compute the r-value for this specific permutation of the data. We repeated this procedure to obtain 10.000 r-values obtained by chance. The participant’s coupling strength was then computed by subtracting the median of the permuted r value distribution from the empirical r value. We then tested whether at the group level, coupling strengths were significantly larger than zero using a one-sided Wilcoxon signed-rank test against zero. Here, we test whether empirical coupling was higher than permuted chance level, and thus one-sided tests were used. Of note, while coupling strength might occasionally be negative (but small) in a given participant, at the group level two signals cannot be significantly less phase-amplitude coupled than what would be expected by chance. Testing the other side of the hypothesis (empirical coupling lower than chance) would thus not be justified.

Page 11, lines 334-348.

** Page 11 | Line 338: The phrase "as they were closest to our research questions" is vague. Please elaborate on the rationale for selecting these two MAIA subscales in relation to the research question. Additionally, it would be informative to include correlation results for the other MAIA subscales.*

We have expanded on the rationale for the inclusion of those two specific MAIA subscales, and explained why the other scales were not tested in the methods section:

For the correlation analysis we focused on the correlations between the coupling strengths and the body listening and noticing sub-scores, which refer to the awareness of comfortable, uncomfortable and neutral bodily signals and active listening to the body for insight respectively, as they were closest to our research questions. The other dimension of the MAIA (Not Distracting, Not Worrying, Attention Regulation, Self-Regulation, and Trusting) were not correlated with coupling scores, as these dimensions mostly relate to attention to pain and discomfort of the body, and the ability to change one’s attention to bodily signals (58), which was not relevant to the current experiment. Correlations with the MAIA questionnaires were FDR corrected for six tests (two sub-scores by three coupling strengths).

Pages 12-13, lines 407-416.

** Page 12 | Line 359: The section title "3.1 Coil distance-to-target explains MEP amplitude" could be rephrased for clarity. Suggested revision: "3.1 Coil-to-target distance explains MEP amplitude."*

Done

** Page 12 | Lines 360-361: The sentence "The distance of the TMS coil to the target stimulation site in the motor cortex is known to influence MEP amplitude (40)." may not be best suited for the Results section. To maintain the section's focus on findings, consider moving this contextual information to the Methods section.*

This information has been moved to the methods section.

** Figure 2D: The visual representation of individuals with the highest coupling strength across organs is unclear. Symbols such as stars and squares may indicate the strongest coupling for one signal but not for others. Please clarify the criteria used to identify these individuals.*

This has now been clarified in the figure 2 legend.

D) Each participant displayed a unique interoceptive profile. To illustrate this inter-individual variability, interoceptive profile of the participant with the largest cardiac-motor coupling (left, red lozenge in C), the participant with the largest respiration-motor coupling (middle, blue star in C), and the participant with the largest gastric-motor coupling (right, green square in C). As can be seen from the bar plots depicting participant’s interoceptive profiles, even though each of these three participants had the highest coupling scores between corticospinal excitability and one of the visceral rhythms, coupling with the two other visceral rhythms was not necessarily large. More profiles are presented in Supplementary Figure 1.

Page 31, lines 1049-1055.

** Page 13 | Line 428: The reference to "Figure 4A, left" appears to be a mislabeling and may actually correspond to Figure 3.*

Figure labels have been corrected.

** Figure 3: Does "MEP amp | distance" refer to MEP residuals? Also, what do the three boxplots per phase (e.g., systole vs diastole) represent? Please clarify.*

We have clarified this in the figure legend.

Exploratory analyses on alignment to cardiac systole/diastole (corresponding to cardiac contraction and relaxation) and inspiration/expiration. A) Mean MEP amplitudes after correction for distance-to-target effects (MEP amp | distance) binned into six phases, reflecting either systole (bins 1-3 with bins spaced equidistantly between 0 and pi) and diastole (bins 4-6 with bins spaced equidistantly between pi and 2pi). The polar histogram (right) indicates the number of participants having their maximal coupling strengths in the bin. In 20 out of 28 participants, cardiac-motor excitability coupling peaked in systole. B) Mean MEP amplitudes corrected for distance-to-target effects (MEP amp | distance) binned into six phases, reflecting either inspiration (bins 1-3 with bins spaced equidistantly between 0 and pi) and expiration (bins 4-6 with bins spaced equidistantly between pi and 2pi). At the group level, MEPs were larger in the early inspiration phase (left), with 17/28 participants having their highest respiratory-motor excitability coupling during inspiration (right). C) Examples of individual data for cardiac (red) and respiratory (blue) coupling, revealing the extent of intersubject variability.

Page 31, lines 1056 - 1066

We have also specified the definition of cardiac systole / diastole in the corresponding section of the Results (3.4 Alignment to cardiac and respiratory phase shows inter-subject variability).

** Page 16 | Lines 489-490: The sentence "We found significant coupling between motor excitability and each of the three rhythms, with similar effect sizes at the group level" seems redundant with the preceding sentence. Consider omitting.*

This sentence has been deleted

** Page 16 | Lines 491-493: The authors present examples of individual variability in coupling across signals. However, it would be valuable to quantify this variability to provide a more robust characterization.*

To give a more transparent overview of individual interoceptive profiles, we have added a supplementary figure 1, in which the interoceptive profiles of all participants are plotted. Furthermore, for each participant we now calculate a variability index. This index has been added to the new figure, and its calculation is explained in the materials and methods:

Lastly, to quantify how flat (i.e., homogeneous profile with similar levels of coupling with all three visceral rhythms) or peaky (i.e., heterogeneous profile with for instance large coupling for one organ and low coupling for the other two), we computed the following variability index (vi) for each participant:

vi = abs(CS_cardiac_-meanCS)+abs(CS_resp_-meanCS)+abs(CS_gastric_-meanCS)

where meanCS is the mean of CS_cardiac_, CS_resp_ and CS_gastric_. Here, larger values indicate more heterogeneous interoceptive profiles and smaller values more homogeneous profiles. This variability index of all participant’s interoceptive profiles is reported in supplementary figure 1.

Page 12, lines 390-399.

And in the supplementary materials:

**Supplementary figure 1.** Individual interoceptive profiles of all 28 participants. For each participant the coupling strength between each of the visceral rhythms (red = cardiac, blue = respiratory, green = gastric) and the amplitude of the MEP is plotted. The variability index (v.i.) of each participant reflects how homogeneous or heterogenous coupling strength was across the different organs, with larger values indicating more heterogeneity. Interoceptive profiles show large variability, with both homogenous (e.g. coupling to all, or to none, of the visceral rhythms) and heterogenous (coupling to one or two, but not all visceral rhythms) profiles.

** Page 16 | Line 497: The phrase "specifying the functional interpretative frameworks on interoception" is unclear. Please clarify what is meant by "functional interpretative frameworks" in this context.*

This sentence has been rewritten

The existence of coupling for all three rhythms despite very different physiological functions and time scales, and the independence of the coupling mechanisms, call for refining and specifying theories offering interpretations on the functional relevance of the impact of visceral rhythms on the brain.

Page 18, lines 577-581.

** Page 16 | Lines 519-520: The phrase "through cranial nerves and spino-thalamic pathways" could be more specific. If referring to the vagus nerve, it should be explicitly named. Otherwise, clarify which other cranial nerves are involved.*

This has now been specified in the sentence.

Firstly, while all three organs are connected to the central nervous systems through cranial nerves (vagal and glossopharyngeal) and spino-thalamic pathways (cardiac, phrenic, and splanchnic nerves), some of those pathways might be organ-specific, as recently shown in mice (59,60). Secondly, each rhythm also has specificities.

Page 19, lines 605-609

** Page 17 | Lines 520-523: The sentence describes how cardiac and respiratory signals may be sensed in the brain. It would also be relevant to briefly mention how descending (efferent) pathways regulate visceral rhythms (cardiac, respiratory, gastric), how they differ across systems, and how they might interact with descending motor pathways.*

Also in line with comments from reviewer 3, we have added a paragraph in the discussion also highlighting existing efferent pathways, and how these might relate to our findings.

Alternatively, cortical motor areas have been shown to control visceral organs, including separate representations of the stomach and diaphragm (73–75) and stimulation of the motor and premotor cortices alters heart rate (76). The location of these representations with respect to the classical or revised (77) motor homunculus is not yet known. Previous work demonstrated that cortical neurons in rat motor cortex influence specifically sympathetic output to the stomach, and suggested colocalization of skeletomotor and sympathetic function within the same cortical region might facilitate coordination of the two functions (78). However, here we find the three visceral phase effects on MEP amplitude within the same stimulation site, which might rather indicate global changes in excitability state of the hand motor representation, rather than viscerotopic effects in M1.

Page 19, line 621-630.

** Page 18 | Lines 572-573: The sentence "acting with different weights in different participants, could account for the large inter-subject variability observed" could be supported by a visual representation. Consider adding a spaghetti plot showing individual coupling strengths across systems (e.g., x-axis: organ; y-axis: strength; one line per participant) to illustrate within-subject patterns.*

We agree with the reviewer that a visual representation of the inter-subject variability is an interesting addition. We have therefore now added a new figure (supplementary figure 1) and legend to make it easier to see the full variability in our sample (see previous comments).

*Reviewer #3: Effects of interoception on cortical processing have been scrutinized in many behavioral  and neuroimaging experiments. The fundamental question remains: whether these effects can represent changes in  the cortical excitability. In the current study the authors addressed this question by investigating effects of three visceral rhythms on the modulation of motor evoked potentials  (MEPs) produced by TMS. They found that MEPs were modulated by all three rhythms, yet the modulation by each rhythm was independent of the modulation by other rhythms.  The study is performed on a very good technical level and is well presented. I have the following comments:

One should carefully check whether changes in the coupling strength (visceral rhythms vs MEPs) between different rhythms can be due to the different signal-to-noise ratios and thus due to the extractability of the phase portrait which affects the estimation of the coupling strength. This would allow in turn to address the question of whether change in the locking is due to the locking itself or due to a definition of the phase (I assume it is particularly relevant for the gastric activity).*

This is indeed a very important point, which was not addressed in the manuscript. We have now clarified for differences in SNR are taken into account by our analysis approach in the methods section.

Because circular spline GLMs allow for only one circular regressor, we thus tested each visceral rhythm separately. Differences in SNR between the different visceral signals (i.e. sensitivity to accurately detect the phase of each rhythm at the time of the pulse) might impact the accuracy of the spline model. However, potential differences in the quality of phase estimation are also taken into account when estimating chance level per participant per visceral rhythm. The final index we used, coupling strength, is thus relatively immune to SNR and phase extraction issues. To correct for the effect of running one test per visceral rhythm, and this potentially inflating p-values, we applied an FDR correction to the obtained p-values.

Page 11, lines 351-358.

*Have authors considered estimating a coupling strength between three visceral rhythms? It might be that the participants with the strongest coupling would have larger co-modulations of MEPs.*

We agree that this is an extremely important question, which given its complexity and for the most part unexplored nature, goes beyond the scope of the current experiment. We have now acknowledged the important of viscera-viscera interactions in the discussion.

Evidence for organ-specific influences on brain signals in humans is still limited. Here, the focus was on excitability changes in the corticospinal motor system, and whether these findings extend to different physiological and cognitive states remains to be tested. Another remaining question is how viscera-viscera interactions moderate effects of visceral rhythms on the brain. Beyond well known-effects of heart rate modulations by the respiratory rhythm (respiratory sinus arrhythmia), most of the effects of viscera-viscera interactions are still unknown. Beyond such effects of phase-rate coupling, there might also be effects of phase-phase and phase-amplitude coupling. The exact nature of these complex interactions, and how they might impact the influence of visceral rhythms on MEPs needs to be addressed in future work.

Page 20, line 630-639.

*It would be desirable to see traces of all three visceral rhythms (are they shown on Fig 1?), particularly gastric activity (over a few cycles). They, of course, can be shown at different times scales.*

We have updated figure 1, to now show traces of data of one representative participant, taken from the same 20 second of recorded data. Additionally we have added a timescale to the figure for clarity.

The figure legend has been updated accordingly.

Experimental set up. Single pulses of TMS were applied to the hand area of the right primary motor cortex with an inter-pulse interval randomized between 6 and 10 seconds. Simultaneously, the neuronavigated coil position (yellow), electrocardiogram (red), respiratory signal (blue), electrogastrogram (green) and electromyography from the left hand (gray) were recorded. Figure shows traces of a 20 second time segment from one participant of the cardiac (raw), respiratory (filtered), gastric (filtered), and EMG (raw) signals. The experimental measure was the Motor Evoked Potential amplitude measured on a hand muscle (first dorsal interosseous), analysed against the phase of the cardiac, respiratory and gastric rhythms. Note that the three rhythms have very different periods (~1s for the heart, ~5 s for respiration, and ~20s for the gastric rhythm). Abbreviations: spTMS, single pulse transcranial magnetic stimulation; ECG, electrocardiogram; EGG, electrogastrogram; EMG, electromyogram; MEP: motor evoked potential.

Page 31, Lines 1027-1036

*The authors write: "These results indicate that independent mechanisms underly the coupling between the cardiac, respiratory and gastric rhythm and motor excitability. " Why would we assume that there should be the same mechanism? What would be the reasons?*

We have clarified in the introduction that the idea of a similar mechanism underlying the three visceral rhythms is mostly an implicit assumption in the field of interoception, which could in theory be possible, but is mostly lacking empirical testing and clear theoretical rationale.

Indeed, it is possible that visceral rhythms exert an influence in a co-dependent manner, for example through global changes in arousal states mediated through the vagus nerve (14). Still, this underlying assumption remains to be empirically tested. In the field of interoception, even if multiple organs are considered they are typically limited to cardiac and respiratory signals (15–19). When it comes to transcranial magnetic stimulation (TMS), only the cardiac rhythm has been measured so far (20–24). To date, no experiment measured all three visceral rhythms, leaving their potential independent influences on the brain speculative.

Page 3-4, lines 79-86.

*What is the rationale for the motor cortex being similarly involved across different interceptive systems? What is known about the motor representation for different visceral systems*

Indeed, efferent influences of motor cortex on visceral organs could also play a role in the current findings, and have now been addressed in the discussion.

Alternatively, cortical motor areas have been shown to control visceral organs, including separate representations of the stomach and diaphragm (73–75) and stimulation of the motor and premotor cortices alters heart rate (76). The location of these representations with respect to the classical or revised (77) motor homunculus is not yet known. Previous work demonstrated that cortical neurons in rat motor cortex influence specifically sympathetic output to the stomach, and suggested colocalization of skeletomotor and sympathetic function within the same cortical region might facilitate coordination of the two functions (78). However, here we find the three visceral phase effects on MEP amplitude within the same stimulation site, which might rather indicate global changes in excitability state of the hand motor representation, rather than viscerotopic effects in M1.

Page 19, lines 621-630.

*The authors write: "MEPs were excluded if their amplitude was below 50mV". This potentially can lead to a bias where very small responses are excluded. You can always calculate min-max difference in this interval similar to what you do for pronounced MEPs.*

We have added a reference to show exclusion of MEPs with a peak to peak amplitude below 50uV is recommended practice, and explained this more specifically in the methods:

MEP amplitude was calculated as the difference between the local maximum and local minimum (peak-to-peak) between 20-60ms after the pulse, falling well within the range in which MEP peaks typically occur (44,45). Following standard practice (46) and in line with previous MEP experiments (e.g. (20,47)), MEPs were excluded if their amplitude was below 50μV. MEPs were furthermore excluded if their amplitude got cut off due to amplifier saturation, or if the signal to noise ratio (SNR) between the time preceding the pulse relative to the MEP amplitude was below three.

Page 7, lines 200-206.

*"Their amplitude got cut off due to amplifier saturation" -- for BrainProducts it can only happen if the amplitude of the AD converter resolution during the acquisition was 0.1, with 0.5 it should work quite well without the saturation. What was the setting during the recordings? And how many MEPs were excluded?*

Indeed the reason there were MEPs where the amplifier saturated is because the first 11 participants, the resolution of the EMG channels was set to 0.1uV/bit, after which this was corrected to 0.5uV, after which the saturation issue no longer occurred. We have added this information now explicitly in the manuscript.

For the first 11 participants, the resolution of the EMG channel was set to 0.1μV per bit, leading to occasional amplifier saturation. In those cases, MEPs were excluded. For the remaining participants, the resolution was set to 0.5μV per bit.

Page 7, lines 207-210.

We have now also added how many trials were on average excluded based on the exclusion criteria of the different signals.

Per participant, a mean(SD) number of trials were excluded due to either MEP amplitude or pre-MEP noise in the MEG (24.6(27.5)), distance-to-target (1.3(1)), cardiac signal (2.5(4.6)), respiratory signal (36.6(37.3=), or gastric signal (14.3(9.8)) (note that a trial was excluded if it failed inclusion criteria for one of the signals, but could be labelled for exclusion due to multiple signals showing issues at once).

Page 9, lines 268-272.

*Since the amplitude of MEPs depends on the pre-pulse EMG, it would be interesting to see whether correlations across subjects were affected by these pre-stimulus measures.*

Indeed pre-pulse EMG influences MEP amplitude, which is why here participants were instructed to keep their hand relaxed during the experiment and any trials containing potential pre-pulse muscle contractions were excluded from analyses. Furthermore, we actively avoided contamination of muscle signals during task responses by having participant’s indicate detected target location using eye-movements. The current dataset is therefore not suitable to examine the influence of the pre-stimulus measures.

*Can you provide a frequency response for the EGG filter?*

Filtering at those low frequencies is indeed tricky, and we rely on previous published work from our group (with associated code) to preprocess and filter the EGG (Wolpert, Rebollo & Tallon-Baudry, *psychophysiology*, 2020), cited in the Material and Methods); Figure 6 panel A in this article shows the filter intended, and actual, frequency profile. We paste it here for your convenience. Note that panels B to F illustrate data that are discarded for lack of a clear spectral peak at several electrodes.
